# Tumor-on-chip and cancer microphysiological platforms: knowledge structure, thematic evolution, and the emergence of organoid-integrated models, 2010–2025

**DOI:** 10.3389/fphar.2026.1926341

**Published:** 2026-09-29

**Authors:** Huichuan Tian, Guanlu Li, Yuheng Lian, Huihui Liu, Yunda Fang, Yao Tong, Ziyi He, Yaoyao Bian, Gang Wei

**Affiliations:** 1 Department of Emergency, Xi’an Hospital of Traditional Chinese Medicine, Xi’an, China; 2 Faculty of Chinese Medicine, Macau University of Science and Technology, Taipa, Macao SAR, China; 3 School of Health Preservation and Rehabilitation, Nanjing University of Chinese Medicine, Nanjing, China; 4 Department of Anorectal Surgery, Xuzhou Hospital of Chinese Medicine Affiliated to Nanjing University of Chinese Medicine, Xuzhou, Jiangsu, China; 5 Jiangsu Provincial Engineering Research Center of TCM External Medication Development and Application, Nanjing University of Chinese Medicine, Nanjing, China

**Keywords:** bibliometric analysis, cancer microphysiological systems, drug screening, microfluidics, precision oncology, tumor microenvironment, tumor-on-chip

## Abstract

**Background:**

Cancer microphysiological systems (MPS), including tumor-on-chip platforms, combine engineered microenvironments, controlled fluid transport, and tumor-relevant biological models for cancer modeling, drug testing, and translational research. Organoid-integrated chips represent one developing component within this broader field, but the publication growth, knowledge structure, and changing research priorities of cancer MPS and tumor-on-chip research have not been systematically mapped across major bibliographic databases.

**Methods:**

We performed a dual-database bibliometric analysis of publications indexed in the Web of Science Core Collection and Scopus from 2010 to 2025. Bibliometric indicators were analyzed using Bibliometrix, VOSviewer, and CiteSpace. We examined publication growth, source distribution, author and country collaboration, highly cited and co-cited references, and temporal keyword evolution. In addition, a phase-split keyword analysis was conducted to compare the research emphasis during 2010–2020 and 2021–2025.

**Results:**

A total of 476 publications were included in the final cancer MPS/tumor-on-chip corpus. Research output increased markedly after 2020 and reached 101 articles in 2025. Source, co-citation, and keyword analyses suggested a progressive shift from early emphasis on microfluidic device construction and physical culture control toward greater attention to biofabrication, tumor microenvironment reconstruction, immune-related interactions, and drug-screening and other application-oriented themes. Phase-split keyword analysis showed that recent publications placed greater attention on biological applications and translational questions while retaining a clear connection with earlier engineering-oriented research. The United States and China emerged as the major research hubs, although with different collaboration profiles.

**Conclusion:**

This study provides a dual-database bibliometric analysis of cancer microphysiological systems and tumor-on-chip research from 2010 to 2025. The field has expanded from microfluidic engineering and basic tumor reconstruction toward tumor microenvironment modeling, multicellular interactions, drug-related applications, and organoid integration. These trends indicate growing translational interest, although the biological performance and clinical utility of individual platforms require direct experimental evaluation.

## 1 Introduction

In the field of oncology research, the development of experimental models that can accurately replicate the physiological and pathological complexities of the human body remains a fundamental challenge for both basic science and clinical translation (Bray et al., 2024). Traditional two dimensional cell culture systems have long served as the primary workhorse for exploring biological mechanisms, yet they are increasingly recognized as inadequate due to their inability to capture the spatial heterogeneity and the intricate extracellular matrix interactions found in native tissues (Diao et al., 2025; Pampaloni et al., 2009). Similarly, while animal models provide a systemic physiological context, their utility is often constrained by significant interspecies differences, high operational costs, and persistent ethical concerns (Edmondson et al., 2014; Fatehullah et al., 2016). These limitations have created a critical gap in our ability to predict drug efficacy and understand tumor progression within a human centric environment. These limitations have motivated the development of cancer microphysiological systems (MPS), including tumor-on-chip platforms, which combine engineered three-dimensional microenvironments with controlled fluid transport and experimental manipulation (Drost and Clevers, 2018; Park et al., 2019). Organoid-integrated chips constitute one biologically complex subgroup within this broader platform family, rather than a synonym for the field as a whole.

Notably, the construction of these platforms has evolved from traditional soft lithography to advanced biofabrication strategies, such as 3D bioprinting and hydrogel-based patterning (Huh et al., 2010; Bhatia and Ingber, 2014; Datta et al., 2020). These platforms leverage the biological fidelity of three dimensional organoids while incorporating the precise environmental control afforded by microfluidic systems. This integration allows researchers to reconstruct the essential elements of the tumor microenvironment on a microscopic scale (Sontheimer-Phelps et al., 2019; Wang et al., 2022; Xie et al., 2021). Unlike static cultures, these chips can simulate dynamic conditions such as interstitial fluid flow, oxygen gradients, and nutrient transport, all of which play decisive roles in tumor growth and therapeutic resistance (Wang et al., 2022; Zervantonakis et al., 2012; Jeon et al., 2015; Lin et al., 2019). The broader organ-on-chip lineage includes the landmark 2010 study by Huh and colleagues, which demonstrated the reconstitution of organ-level lung functions on a chip (Huh et al., 2010). Although this study was neither a tumor nor an organoid study, it provided an important conceptual and engineering foundation for subsequent tissue-on-chip and tumor-on-chip applications. Since then, driven by innovations in biomaterials and bioassembly, the field has transitioned from basic, lithography-based microchannel designs to integrated systems that incorporate multiple cell types, including immune cells and vascular endothelial cells, to better mimic the chaotic nature of cancer.

The clinical implications of this technology are particularly significant in the context of precision medicine and personalized oncology (Tong et al., 2024; Chitrangi et al., 2023; Ayuso et al., 2022). In a subset of cancer MPS studies, patient-derived organoids, tumor spheroids, primary tumor tissues, or engineered tumor constructs are incorporated into microfluidic environments to evaluate treatment responses under controlled experimental conditions (Tuveson and Clevers, 2019; Yi et al., 2019; Li et al., 2021). These models may provide information that is not readily captured by conventional monolayer cultures, but their use for clinical decision-making remains an emerging possibility because analytical validation, inter-laboratory reproducibility, prospective clinical correlation, and standardized reporting are still incomplete. Such personalized models have the potential to significantly improve the predictability of clinical outcomes and reduce the time required for drug selection (Pradhan et al., 2018; Rodriguez et al., 2020; Frankman et al., 2022). Furthermore, advanced platforms are now being designed to simulate the systemic metabolism of drugs and their off target toxicities, potentially offering a viable alternative to traditional animal testing in the pharmaceutical industry (Roy and Cucullo, 2026; Cao et al., 2026). As the research in this domain expands, the focus is shifting toward the inclusion of more sophisticated biological components, such as the recruitment of fibroblasts and the activation of immune pathways, to address the remaining challenges in model fidelity (Ch et al., 2026; Kast et al., 2026).

The rapid expansion and increasing heterogeneity of cancer MPS and tumor-on-chip research now make it difficult to assess the field through narrative reading alone. The literature spans multiple model classes, including tumor cell-line systems, spheroids, organotypic tissues, patient-derived models, vascularized constructs, and organoid-integrated platforms. It also combines distinct engineering and biological dimensions, such as microfluidic architecture, biomaterials, perfusion, extracellular matrix design, immune or stromal incorporation, biosensing, and pharmacological testing. Bibliometric analysis can complement conventional reviews by quantitatively mapping publication growth, source distribution, collaboration structures, influential references, and the temporal emergence of research themes.

Recent reviews and bibliometric studies have examined organoid models, tumor-on-chip systems, vascularized tumor platforms, and microfluidic approaches to cancer modeling from partially overlapping perspectives (Gonçalves et al., 2022; Huang and Tu, 2023; Trujillo-de Santiago et al., 2019). These studies provide important descriptions of individual technologies and applications, but the knowledge structure of the broader cancer MPS and tumor-on-chip field, including its engineering foundations, biological model classes, translational applications, and emerging organoid integration, remains insufficiently synthesized across databases. In particular, the relationship between early microfluidic and tissue-engineering research and more recent emphasis on tumor microenvironment reconstruction, immune-related interactions, drug screening, and patient-derived models has not been systematically examined as a temporal knowledge trajectory.

In this context, the present study conducts a dual-database bibliometric analysis of cancer MPS and tumor-on-chip research indexed in the Web of Science Core Collection and Scopus from 2010 to 2025. We characterize publication growth, source distribution, author and affiliation-entity contributions, international collaboration, highly cited publications, co-cited references, keyword co-occurrence, thematic clustering, citation bursts, and temporal topic evolution. We further compare the 2010–2020 and 2021–2025 periods to examine changes in research emphasis from platform engineering and microfluidic control toward tumor microenvironment reconstruction, drug screening, immune-related modeling, and other translational applications. Organoid-integrated models are interpreted as an emerging component of this broader field rather than as an eligibility requirement for every included publication. The objective is therefore to clarify the knowledge structure and thematic evolution of cancer MPS and tumor-on-chip research while identifying the growing role of organoid integration within this landscape.

## 2 Materials and methods

### 2.1 Data sources and search strategy

The bibliographic data for this study were systematically retrieved from the Web of Science Core Collection (WoSCC) and the Scopus database to ensure a comprehensive and multidimensional evaluation of the MPS and tumor-on-chip research. Based on the predefined search protocol, the query utilized for WoSCC was: TS = (Neoplasms OR Neoplasia OR Tumor OR Cancer OR Malignancy OR “Benign Neoplasm”OR “Tumor Organoid” OR “Cancer Organoid” OR “Patient-derived Organoid” OR Tumoroid) AND TS = (“organoid on a chip” OR “organoid chip” OR “Microfluidic organoid platform” OR “tissue on a chip” OR “tumor on a chip” OR “cancer on a chip” OR “organoid microdevice” OR “bioengineered organoid chip system” OR “microphysiological systems” OR “tumor-on-chip” OR “3D microfluidic models”)

Similarly, for the Scopus database, the search string was: TITLE-ABS-KEY (Neoplasms OR Neoplasia OR Tumor OR Cancer OR Malignancy OR “Benign Neoplasm”OR “Tumor Organoid” OR “Cancer Organoid” OR “Patient-derived Organoid” OR Tumoroid) AND TITLE-ABS-KEY (“organoid on a chip” OR “organoid chip” OR “Microfluidic organoid platform” OR “tissue on a chip” OR “tumor on a chip” OR “cancer on a chip” OR “organoid microdevice” OR “bioengineered organoid chip system” OR “microphysiological systems” OR “tumor-on-chip” OR “3D microfluidic models”).

To maintain the rigor of the analysis and ensure high academic quality, the document types were strictly limited to original research articles and review papers, while excluding meeting abstracts, letters, and editorials. The search was conducted on 3 January 2026, in order to maximize coverage of publications from 2025. Records indexed as 2026 publications were excluded to maintain a consistent publication window of 2010–2025 (Figure 1). Because the retrieval strategy was designed to maximize sensitivity to the heterogeneous cancer MPS and tumor-on-chip literature, a prespecified secondary scope-consistency screening was performed after database merging and initial relevance screening. This additional step was used to distinguish cancer-related MPS and tumor-on-chip studies from non-oncologic organ-chip research, generic engineering studies without a cancer application, and publications with only incidental references to cancer or organoid technology.

**FIGURE 1:**
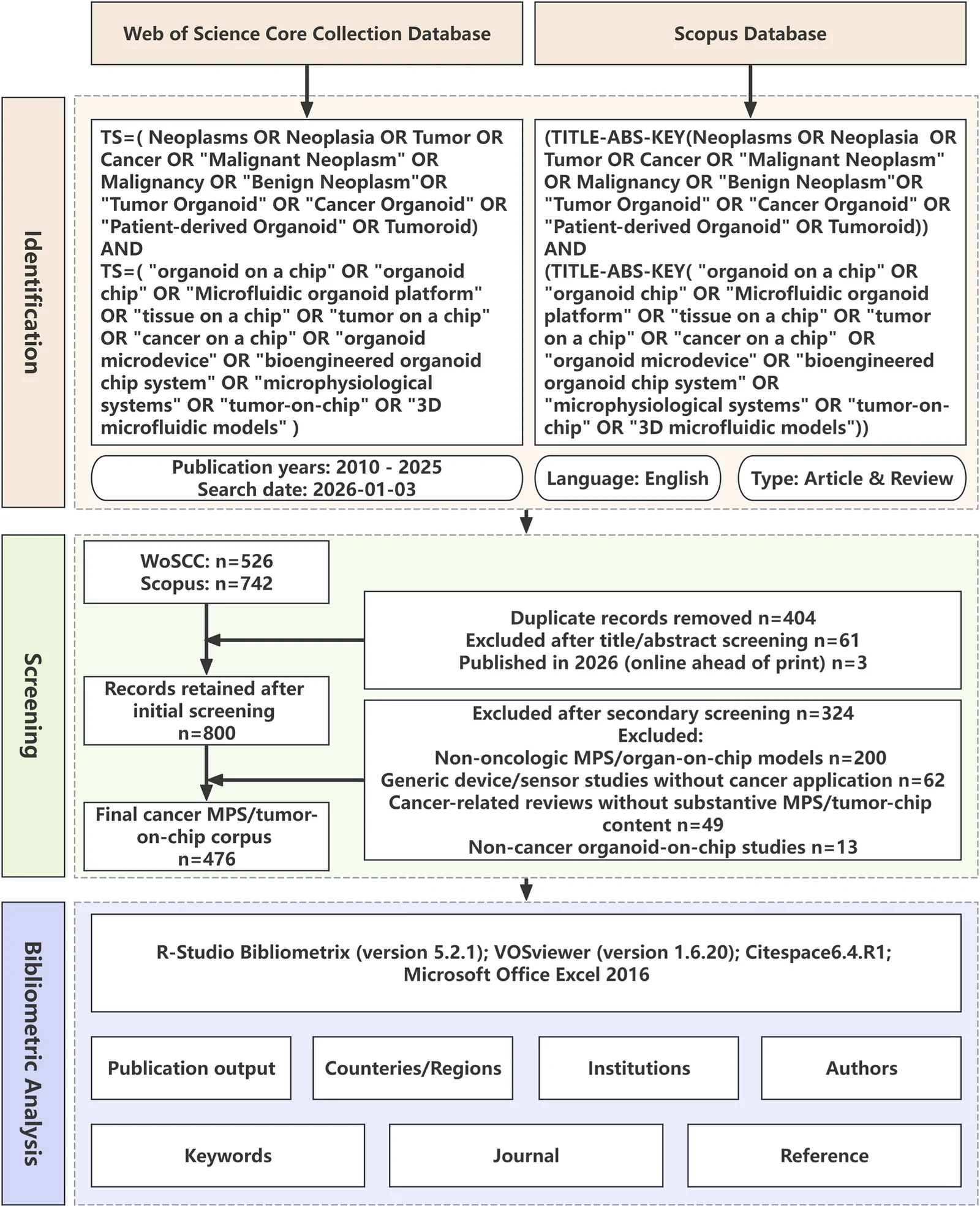
Study selection and bibliometric analysis workflow. Flow chart illustrating data retrieval from the Web of Science Core Collection and Scopus, application of eligibility criteria, duplicate removal, title/abstract screening for topical relevance, secondary scope-consistency screening, exclusion of 324 records, and final inclusion of 476 publications, and the subsequent bibliometric analyses performed in this study.

### 2.2 Data preprocessing and bibliometric analysis

Following retrieval, full bibliographic records and cited references were exported from the Web of Science Core Collection and Scopus in plain text and CSV formats. The records from the two databases were imported into the Bibliometrix package (version 5.2.1) in R for preprocessing and bibliometric analysis. Duplicate records were identified using the mergeDbSources function and were further manually checked based on DOI and title information to ensure the consistency of the final dataset. The remaining records were further screened based on titles and abstracts for topical relevance. During secondary title/abstract screening, records were excluded when they did not address cancer-related microphysiological systems (MPS) or tumor-on-chip platforms. Specifically, we excluded (i) non-oncologic organ-on-chip or MPS studies; (ii) generic device, material, sensor, or fabrication studies without a substantive cancer application; (iii) cancer-related reviews that only mentioned MPS or tumor-on-chip concepts without substantive discussion; and (iv) non-cancer organoid-on-chip studies. Of the 800 records retained after the initial screening, 324 were excluded during this scope-consistency screening, leaving 476 publications in the final corpus. The cleaned dataset was used to calculate the main bibliometric indicators, including annual publication output, average citations per document, source productivity, author productivity, institutional contributions, country-level output, total citations, local citations, H-index, G-index, M-index, and collaboration link strength. Co-authorship, co-citation, and keyword co-occurrence networks were visualized using VOSviewer (version 1.6.20) and CiteSpace (version 6.4.R1). Microsoft Excel 2016 was used for data organization and descriptive plotting.

For institution- and country-level collaboration analyses, affiliation data were analyzed according to the indexed entity names provided by the source databases. Therefore, system-level entities, university systems, national research organizations, and single-campus institutions were retained as distinct affiliation entities when they appeared as such in the original records. The institutional analysis was intended to describe collaboration patterns among indexed affiliation entities rather than to generate a fully normalized ranking of independent universities.

### 2.3 Phase-split keyword analysis and visualization

To further characterize the MPS and tumor-on-chip research, a phase-split analysis was performed based on the temporal distribution of annual publications and keyword evolution. The study period was divided into an earlier research exploration phase (2010–2020) and a later expansion phase (2021–2025). The 2021 boundary was selected as a pragmatic analytical division because publications from 2021 to 2025 accounted for 77.1% of the final corpus and represented the period of sustained expansion observed in the dataset. This division was not intended to define a statistically determined breakpoint or to imply a complete discontinuity between the two periods. The same cleaned dataset was then divided into two time-defined subsets for independent keyword-based analyses.

Keyword co-occurrence, clustering, timeline visualization, and citation-burst analyses were conducted using the author-supplied and database-indexed keywords. Synonymous terms were harmonized before analysis. Network and density maps were generated using VOSviewer, whereas clustering, timeline, and burst analyses were performed in CiteSpace. For the full study period, the CiteSpace analysis covered 2010–2025 with 1-year slices. The selection criterion was the g-index with k = 25, together with LRF = 2.5, L/N = 10, LBY = 5, and e = 1.0. No pruning was applied. The phase-split analyses used the same analytical framework and were conducted separately for 2010–2020 and 2021–2025. For thematic trend analysis, all keywords were included from 2010 to 2025, with a minimum word frequency of five and three keywords displayed per year.

Burst keyword detection was conducted separately for the two phases to identify phase-specific emerging topics. The burst strength and duration were used to estimate the relative prominence and persistence of each keyword. Timeline maps were generated to compare the chronological organization of thematic clusters between the two phases. This phase-split design enabled the distinction between early engineering-oriented studies and the later expansion toward biologically complex and application oriented research.

## 3 Results

### 3.1 Annual publications and average citations

A total of 476 publications were included in the final MPS and tumor-on-chip corpus, covering the period from 2010 to 2025. Publication activity remained limited during the early years, with no records identified in 2011 or 2012. A clear increase became evident after 2018, and the annual output continued to rise despite a modest fluctuation in 2022. The number of publications reached 101 in 2025, the highest level observed during the study period. Notably, 367 publications were published between 2021 and 2025, accounting for 77.1% of the final corpus. These findings indicate that the field has entered a period of sustained expansion, with most of its indexed literature emerging during the later years of the study period (Figure 2).

**FIGURE 2:**
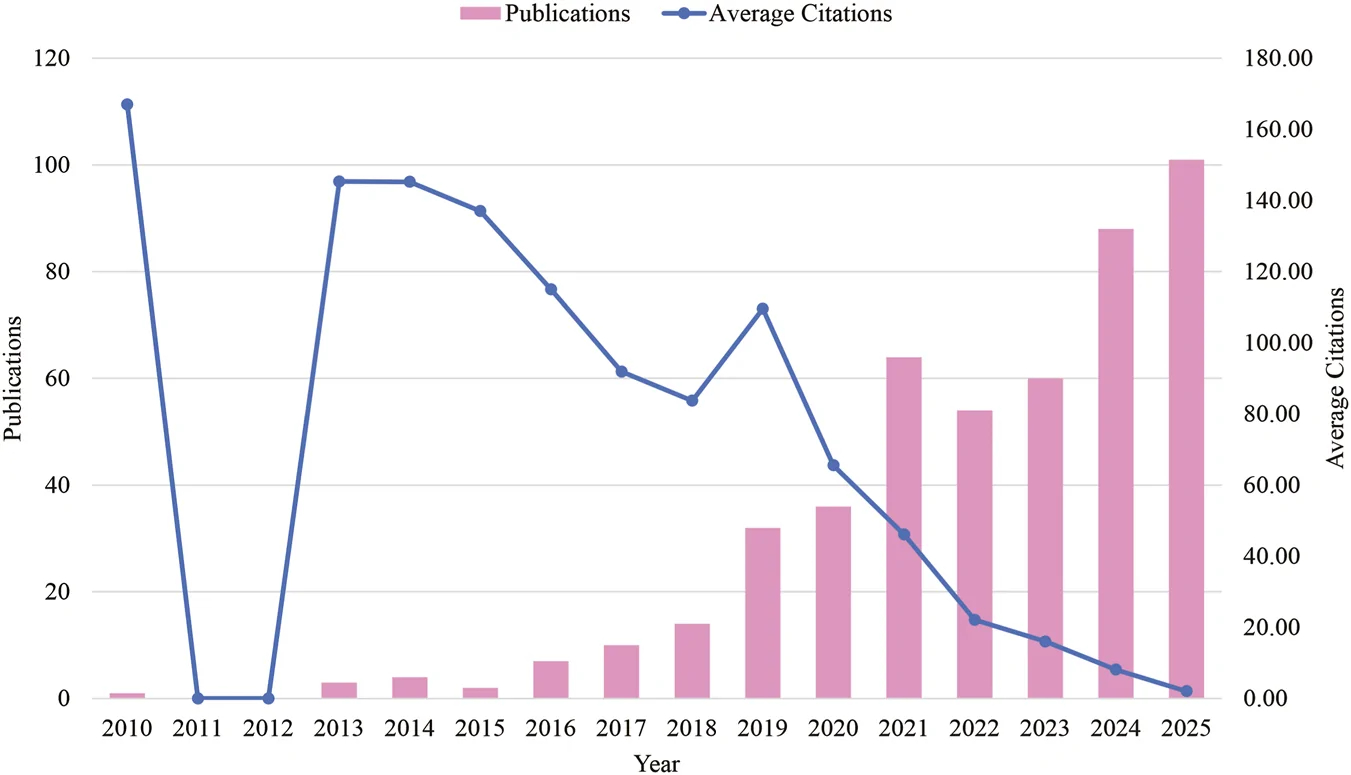
Annual publication output and mean citation count in MPS and tumor-on-chip research from 2010 to 2025. The bars represent the annual number of publications, and the line represents the mean citations per article based on the citation data retrieved for this study.

The annual citation pattern differed from the publication trend. Early publications had relatively high mean citation counts, reaching 167 citations per article in 2010 and approximately 145 citations per article in both 2013 and 2014. A temporary increase was also observed in 2019, when the mean citation count reached 109.59 per article. Citation averages declined in the subsequent years, with the lowest value observed in 2025. This decline should be interpreted cautiously because publications from the most recent years have had a much shorter time to accumulate citations. Thus, the citation pattern reflects both the visibility of earlier publications and the unequal citation windows across publication years, rather than a direct change in the scientific value of the field.

### 3.2 Sources

The top journals in the field of MPS and tumor-on-chip research were identified based on their publication volume and citation impact. Among these, *“LAB ON A CHIP”* emerged as the most productive and influential source by publishing 29 articles with the highest H-index of 16 and G-index of 29. It also received the highest number of total citations, with 1,618 citations, indicating its prominent role as a publication and citation venue for microfluidic engineering and lab-on-a-chip research. “CANCERS” and “ADVANCED HEALTHCARE MATERIALS” followed with 27 and 24 publications, respectively, reflecting the close connection between tumor biology, biomaterials, and microfluidic platform development.

Analysis of impact factors reveals that *“ADVANCED DRUG DELIVERY REVIEWS” *holds the highest impact (21.0), followed by “SMALL” (11.8) and *“ADVANCED HEALTHCARE MATERIALS”* (11.0). Although the number of publications varied across journals, several sources received substantial citation attention within the 476-record corpus, including the *“INTERNATIONAL JOURNAL OF MOLECULAR SCIENCES”* with 1,117 citations and *“SCIENTIFIC REPORTS”* with 724 citations. These metrics suggest that the literature is mainly distributed across journals covering bioengineering, microsystems, biomedical materials, tumor biology, and drug delivery (Table 1).

**TABLE 1:** Top 10 sources in the research field of MPS and tumor-on-chip research.

Journal	Publications	h_index	g_index	Total citations	IF(2025)
Lab on a chip	29	16	29	1618	6.3
Cancers	27	14	27	741	4.8
Advanced healthcare materials	24	12	24	693	11.0
Biofabrication	13	5	9	88	8.2
Scientific reports	12	8	12	724	4.9
Frontiers in bioengineering and biotechnology	11	7	11	178	5.8
International journal of molecular sciences	11	8	11	1117	5.6
Small	10	7	10	408	11.8
Micromachines	9	5	9	201	3.5
Advanced drug delivery reviews	8	8	8	402	21.0

As shown in Figure 3A, *LAB ON A CHIP, CANCERS*, and *ADVANCED HEALTHCARE MATERIALS* occupied the leading positions in publication output, while the h-index and g-index reflected differences in citation performance among the major sources. The results also indicate that publication productivity and citation visibility were not completely concordant across journals. Some sources with a relatively modest number of publications, such as the *INTERNATIONAL JOURNAL OF MOLECULAR SCIENCES* and *SCIENTIFIC REPORTS*, nevertheless received substantial citation attention within the present corpus. The journal co-citation network and density map (Figures 3B,C) further showed that the literature was connected through journals covering bioengineering, biomedical materials, microfluidics, tumor biology, and oncology. *LAB ON A CHIP, ADVANCED HEALTHCARE MATERIALS, CANCERS*, and BIOFABRICATION occupied relatively visible positions in this network, consistent with their publication output and citation activity. Figure 3D illustrates the annual publication trajectories of the top 10 journals and shows that most of these sources became more active during the later years of the study period, particularly after 2020. The journal dual-map overlay (Figure 4) provided an additional view of the disciplinary relationships underlying the field. Journals on the citing side were distributed across molecular biology, immunology, medicine, physics, materials science, and chemistry, whereas the cited side included journals associated with molecular biology, genetics, medicine, and related materials disciplines. The citation paths between these categories suggest that cancer MPS and tumor-on-chip research has developed through the interaction of engineering-based platform design with biological and medical research. This distribution is consistent with a field in which microfluidic systems, biomaterials, tumor biology, and translational applications are closely connected within the same knowledge structure.

**FIGURE 3:**
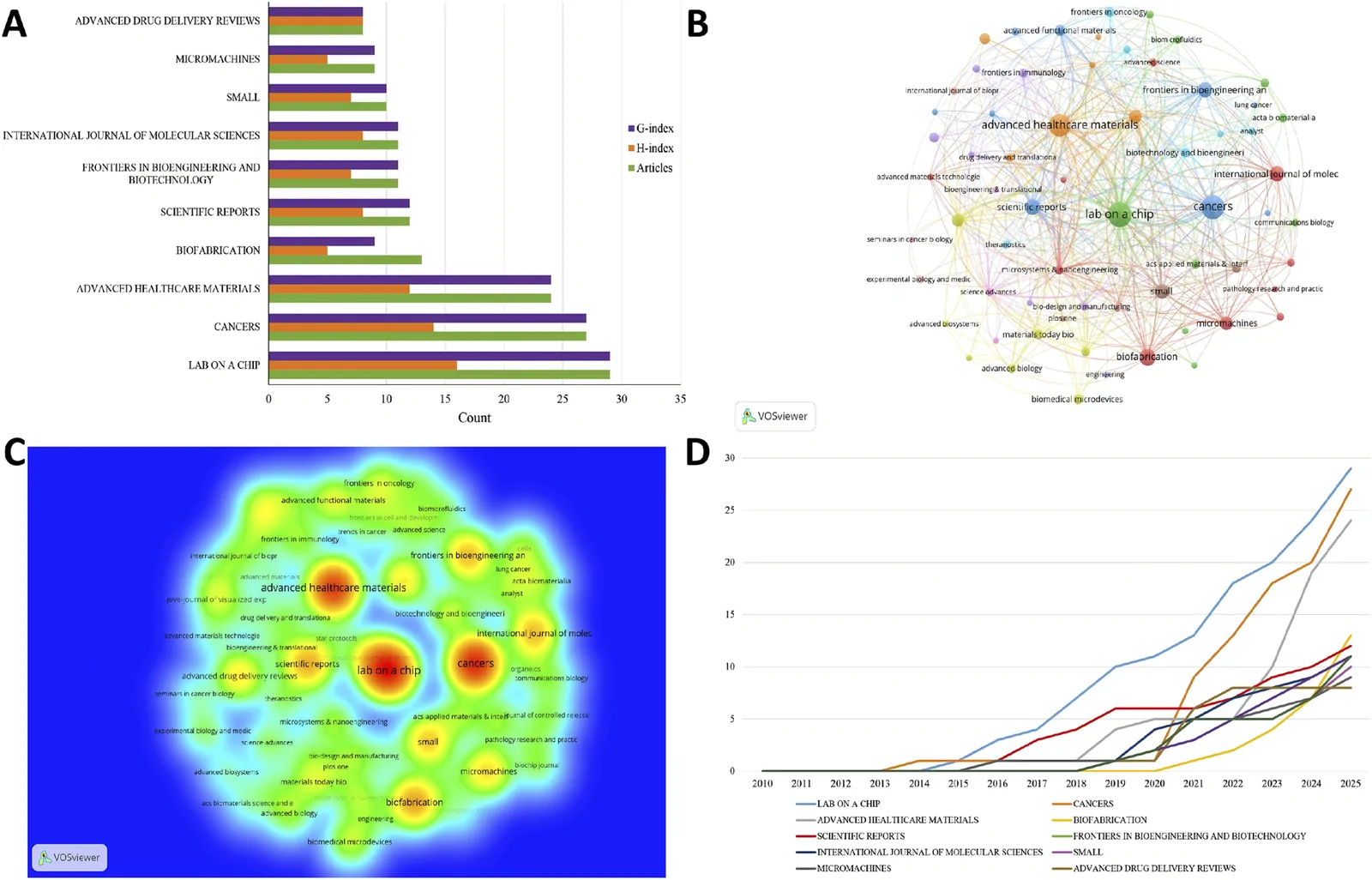
Influential journals in the research field of MPS and tumor-on-chip research. **(A)** Publication volume, H-index, and G-index of the top 10 journals. **(B)** Co-citation network map of influential journals. **(C)** Density map of influential journals. **(D)** Annual publication volume of the top 10 journals.

**FIGURE 4:**
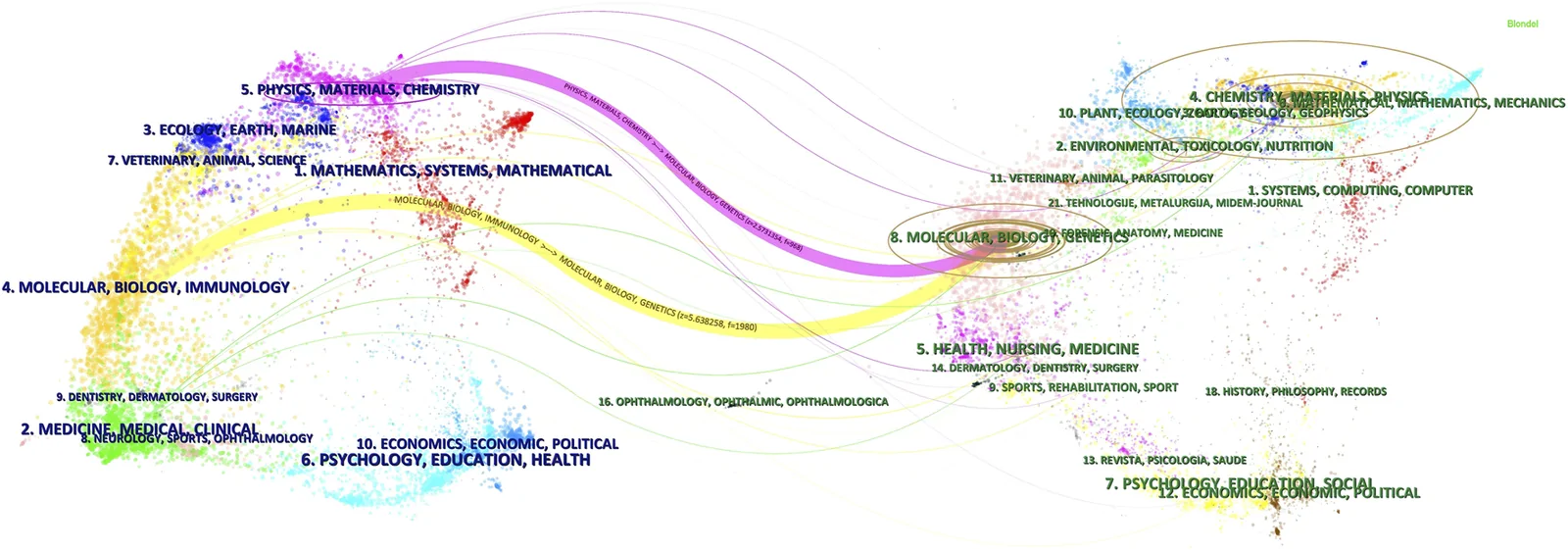
Dual-map overlay analysis.

### 3.3 Author

A total of 135 authors met the threshold for inclusion in the co-authorship analysis. Christopher C. W. Hughes as the most productive author, with 10 documents, followed by Zhang Yu Shrike and Stephanie Descroix, with nine documents each. Steven C. George received the highest number of citations (869), followed by Hughes (703) and Zhang Yu Shrike (668). Stephanie J. Hachey also received considerable citation attention, with 538 citations from seven documents. These results indicate that several active research groups have contributed substantially to the development of MPS and tumor-on-chip research.

The structural characteristics of the author community are illustrated in Figure 5 through co-authorship, productivity, and density analyses. The co-authorship network (Figure 5A) shows several research clusters rather than a single uniformly connected collaboration network. Mechta-Grigoriou, Parrini, Descroix, and Zalcman were located within a closely connected cluster, whereas Hughes, George, and their collaborators formed another visible group. Figure 5B compares publication output with total citations among the leading authors, and Figure 5C provides a density view of the more active collaborative areas in the network. Overall, the author network suggests that cancer MPS and tumor-on-chip research has developed through contributions from several groups working across microfluidics, biomaterials, tumor biology, and translational modeling.

**FIGURE 5:**
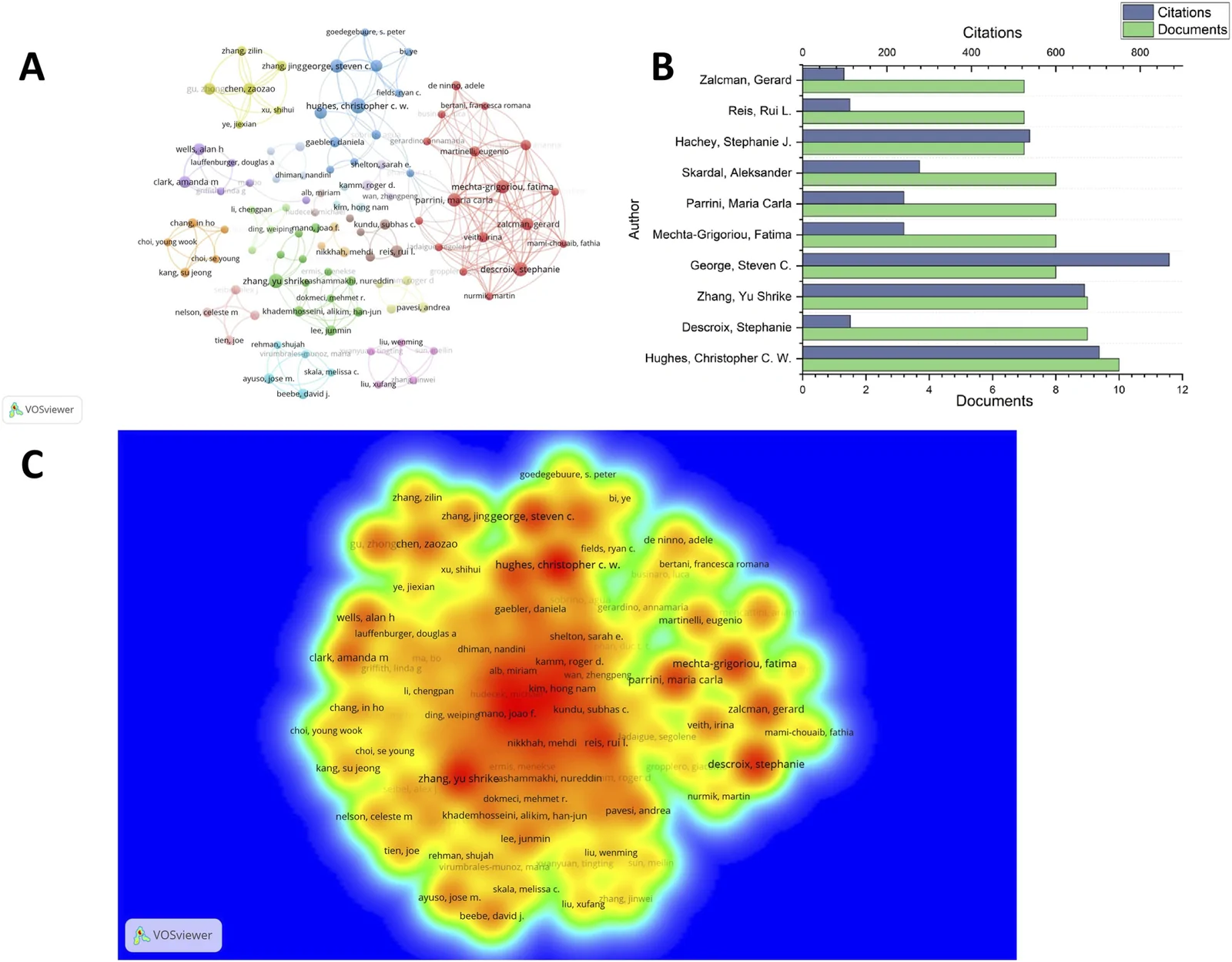
Author collaboration and productivity in MPS and tumor-on-chip research. **(A)** Co-authorship network map of influential authors. **(B)** Publication volume and total citations of the top 10 authors. **(C)** Density map of author collaboration and influence.

### 3.4 Regional distribution

To characterize collaboration patterns at the institutional level, we analyzed the affiliation entities indexed in the source databases. These entities included university systems, national research organizations, and individual institutions, and were therefore treated as database-level affiliation units rather than fully normalized standalone universities. The University of California System ranked first with 67 articles, followed by Institut Curie with 39 articles and the University of Pittsburgh with 31 articles. Other prominent indexed affiliation entities included the Pennsylvania Commonwealth System of Higher Education (30 articles), Harvard University (29 articles), UNICANCER (29 articles), and the Centre National de la Recherche Scientifique (27 articles). The top 10 indexed affiliation entities by publication output are presented in Table 2.

**TABLE 2:** Top 10 indexed affiliation entities by publication output in MPS and tumor-on-chip research.

Institution	Country	Articles
University of california system	USA	67
Institut curie	France	39
University of pittsburgh	USA	31
Pennsylvania commonwealth system of higher education (pcshe)	USA	30
Harvard university	USA	29
Unicancer	France	29
Centre national de la recherche scientifique (cnrs)	France	27
University of wisconsin system	USA	27
Universite psl	France	25
University of texas system	USA	25

Among all countries conducting research in this field, the United States and China had the highest publication outputs, with 148 and 70 articles. South Korea followed with 38 articles, while Italy, the United Kingdom, France, and the Netherlands contributed 20, 19, 17, and 16 articles, respectively. The Single-country publications (SCP) and multiple-country publications (MCP) distribution in Figure 6A shows that publications from the United States and China were mainly single-country publications, with 124 SCP and 24 MCP for the United States and 60 SCP and 10 MCP for China. France and the Netherlands showed a relatively higher proportion of MCPs, with nine of 17 French publications and seven of 16 Dutch publications involving multiple countries. The country collaboration network in Figure 6B places the United States and China near the center of the international collaboration structure, while the world map and density map in Figure 6C,D show that publication activity was concentrated mainly in North America, East Asia, and Europe.

**FIGURE 6:**
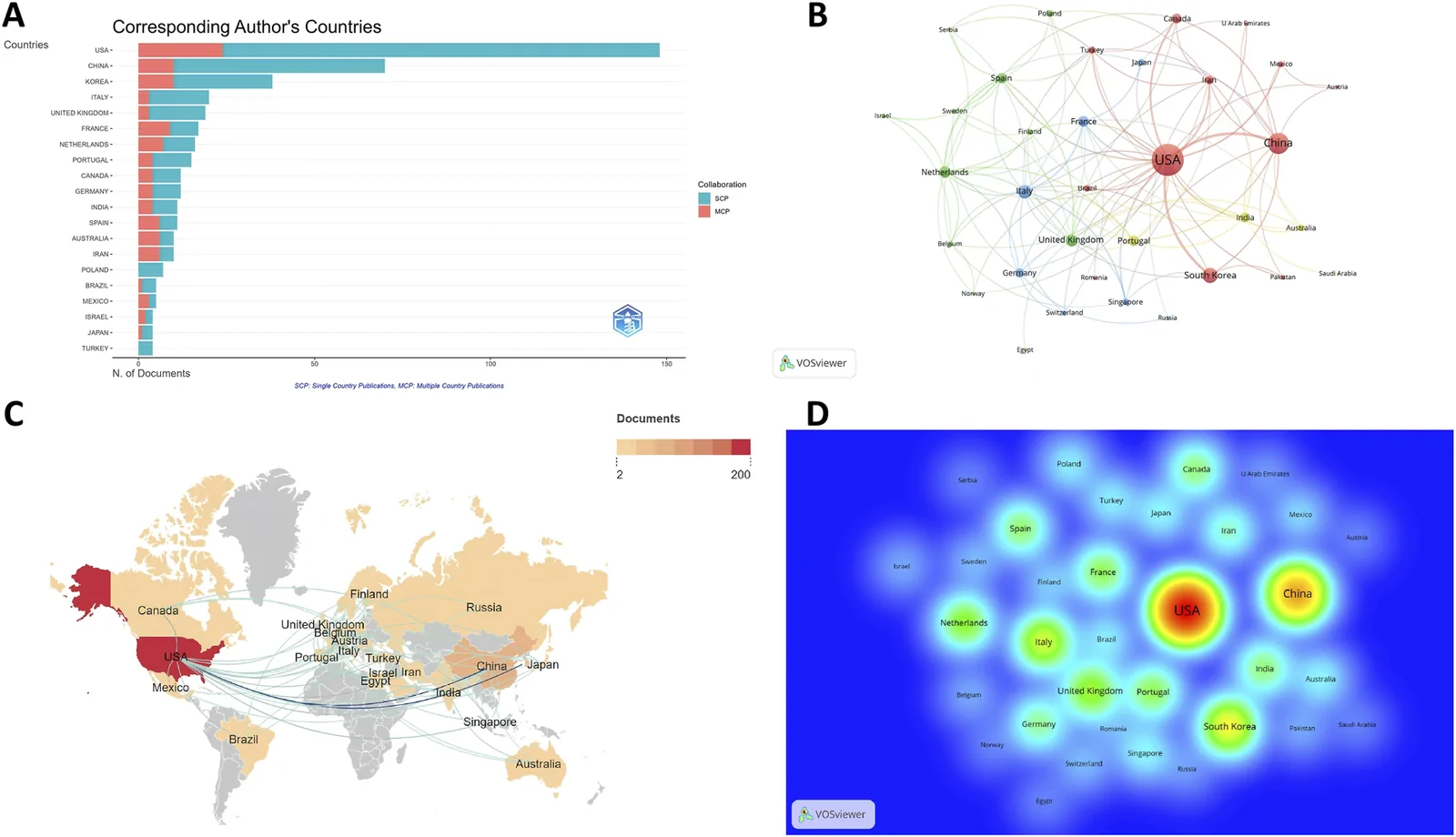
Country-country collaboration in the research field of MPS and tumor-on-chip research. **(A)** SCP and MCP of the top 10 countries. **(B)** Country collaboration network map. **(C)** World map of publication output by influential countries. **(D)** Country collaboration density map.

The regional distribution also showed differences in publication output and collaboration structure. The United States remained the main contributor in the dataset, whereas China and South Korea formed other major centers of publication activity. European countries, including Italy, the United Kingdom, France, the Netherlands, and Portugal, contributed a smaller but clearly visible share of the literature and were connected with a relatively broad international collaboration network. These findings indicate that research activity is geographically concentrated, while the collaboration links shown in Figure 6B,C extend across North America, Asia, and Europe.

Overall, the regional analysis showed uneven publication productivity together with distinct patterns of institutional and international collaboration in cancer microphysiological systems and tumor-on-chip research. The affiliation-entity analysis identified several prominent university systems, research organizations, and individual institutions, whereas the country-level analysis placed the United States and China at the center of the global publication and collaboration network. These results provide a structural overview of the field before the subsequent citation and co-citation analyses of influential publications and references.

### 3.5 Citation analysis of articles and references

For the included publications, citation-based analyses were conducted at both the article and reference levels. At the article level, total citation indicators were used to identify the most highly cited papers in the field and to visualize their citation relationships. At the reference level, co-citation analysis was performed to identify the most influential cited references and to characterize the intellectual base underlying MPS and tumor-on-chip research.

At the article level, the review by Roma-Rodrigues C. published in 2019 in the International Journal of Molecular Sciences was the most highly cited publication in the corpus, with 952 citations (Roma-Rodrigues et al., 2019). Other highly cited publications included the study by Yi H. on a bioprinted glioblastoma-on-a-chip (Yi et al., 2019), the microfluidic breast cancer model reported by Bersini S., and studies by (Roma-Rodrigues et al., 2019; Bersini et al., 2014; Gunti et al., 2021; Nashimoto et al., 2020). These publications covered tumor microenvironment research, organoid and spheroid modeling, vascularized tumor platforms, and drug-response evaluation. The results therefore point to several interconnected lines of work rather than a single publication type dominating the field. In particular, the high citation counts of the Roma-Rodrigues review and the Gunti review should be understood in relation to their broad conceptual and methodological coverage, whereas studies such as those by Yi, Nashimoto and Shirure were more directly related to engineered tumor models and chip-based applications (Figure 7A; Table 3).

**FIGURE 7:**
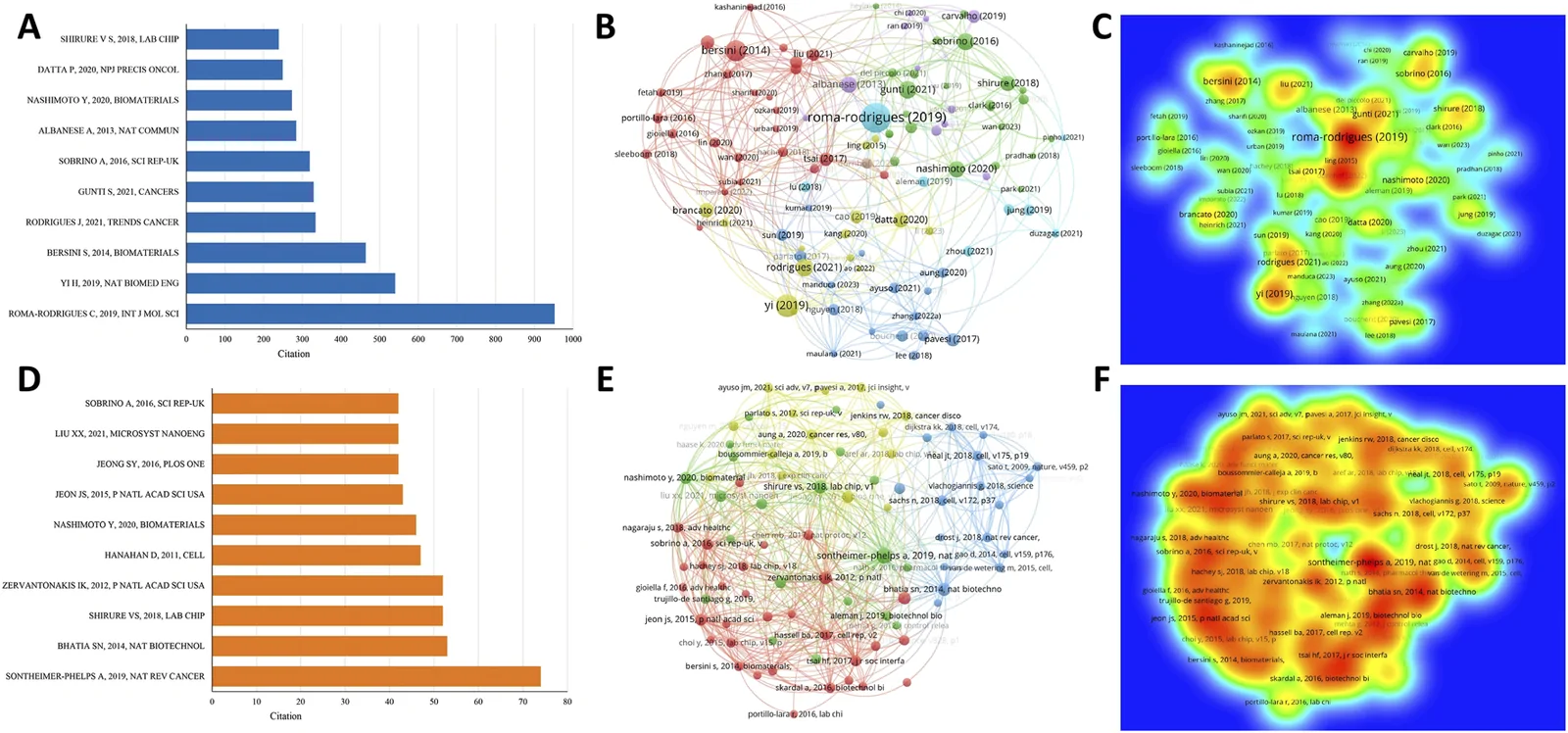
Citation and co-citation analysis of publications and references in MPS and tumor-on-chip research. **(A)** Top 10 globally cited publications. **(B)** Citation network of highly cited publications. **(C)** Citation density map. **(D)** Top 10 co-cited references. **(E)** Co-citation network of cited references. **(F)** Co-citation density map.

**TABLE 3:** Top 10 globally cited publications in MPS and tumor-on-chip research.

Paper	Total citations	TC per year	Normalized TC
Roma-rodrigues et al., 2019, int j mol sci	952	119.00	8.69
Yi et al., 2019, nat biomed eng	540	67.50	4.93
Bersini et al., 2014, biomaterials	464	35.69	3.19
Rodrigues et al., 2021, trends cancer	334	55.67	7.24
Gunti et al., 2021, cancers	329	54.83	7.13
Sobrino et al., 2016, sci rep-UK	319	29.00	2.77
Albanese et al., 2013, nat commun	284	20.29	1.95
Nashimoto et al., 2020, biomaterials	273	39.00	4.16
Datta et al., 2020, npj precis oncol	249	35.57	3.80
Shirure et al., 2018, lab chip	239	26.56	2.85

The citation network and density map (Figures 7B,C) showed that the highly cited publications were connected through several research themes. Roma-Rodrigues, Yi, Bersini, Nashimoto, Sobrino, and Shirure were located within the broader citation structure linking tumor microenvironment modeling, vascularized microfluidic systems, three-dimensional tumor models, and drug screening (Yi et al., 2019; Roma-Rodrigues et al., 2019; Bersini et al., 2014; Gunti et al., 2021; Nashimoto et al., 2020; Sobrino et al., 2016). This pattern is consistent with the multidisciplinary development of MPS and tumor-on-chip research, in which conceptual reviews, engineering studies, and application-oriented models are cited together. The citation density map further showed that the strongest citation activity was concentrated around a limited number of highly visible publications, although citation density should be interpreted as a measure of scholarly attention rather than direct evidence of experimental or clinical superiority.

At the reference level, co-citation analysis identified Sontheimer-Phelps et al. (2019) as the most frequently co-cited reference, with 74 citations from the present corpus. It was followed by Bhatia and Ingber (2014), Shirure et al. (2018), and Zervantonakis et al. (2012), each of which received more than 50 co-citations. Other frequently co-cited references included Hanahan and Weinberg (2011), Nashimoto et al. (2020), Jeon et al. (2015), Jeong et al. (2016), Liu et al. (2021), and Sobrino et al. (2016), (Figure 7D; Table 4). The co-citation network and density map (Figures 7E,F) placed these references within a shared intellectual structure involving organ-on-chip engineering, tumor microenvironment reconstruction, vascularized tumor models, cancer biology, and drug-response assessment. The presence of general cancer and organ-on-chip references, including Hanahan and Weinberg (2011) and Huh et al. (2010), indicates that the field continues to draw on broader conceptual and engineering foundations in addition to cancer-specific chip studies. These results characterize the knowledge base of the wider cancer MPS and tumor-on-chip field and should not be interpreted as evidence that every cited reference concerns organoid-integrated models.

**TABLE 4:** Top 10 co-cited references in MPS and tumor-on-chip research.

Cited reference	Citations	Total link strength
Sontheimer-phelps et al., 2019, nat rev cancer, v19, p65	74	914
Bhatia et al., 2014, nat biotechnol, v32, p760	53	618
Shirure et al., 2018, lab chip, v18, p3687	52	661
Zervantonakis et al., 2012, p natl acad sci USA, v109, p13515	52	652
Hanahan et al., 2011, cell, v144, p646	47	462
Nashimoto et al., 2020, biomaterials, v229, p0	46	613
Jeon et al., 2015, p natl acad sci USA, v112, p214	43	612
Jeong et al., 2016, plos one, v11, p0	42	634
Liu et al., 2021, microsyst nanoeng, v7, p0	42	443
Sobrino et al., 2016, sci rep-UK, v6, p0	42	610

Taken together, the article-level citation results highlight the most visible and influential publications within the field, whereas the reference-level co-citation results reveal the deeper intellectual framework on which the field has been built. This dual perspective helps distinguish between papers that are highly recognized in the literature and references that serve as the enduring conceptual and methodological foundations of MPS and tumor-on-chip research.

Reference clustering and citation-burst analysis provided a further view of the themes that have attracted attention within MPS and tumor-on-chip research. The clusters shown in Figure 8A included “on-chip microplatform,” “living organoid biobank,” “cancer medicine,” “translational tool,” “intercellular interaction,” “on-chip technology,” “*in vitro* tumor model,” “metastatic cascade,” and “perfused tumor.” Together, these labels suggest that the cited literature covers both the engineering development of chip-based platforms and their application to tumor modeling, metastasis research, drug evaluation, and organoid-related studies. The presence of “living organoid biobank” and “translational tool” among the clusters is consistent with the growing interest in linking engineered models with patient-relevant biological materials, although the clustering results alone do not establish clinical validity.

**FIGURE 8:**
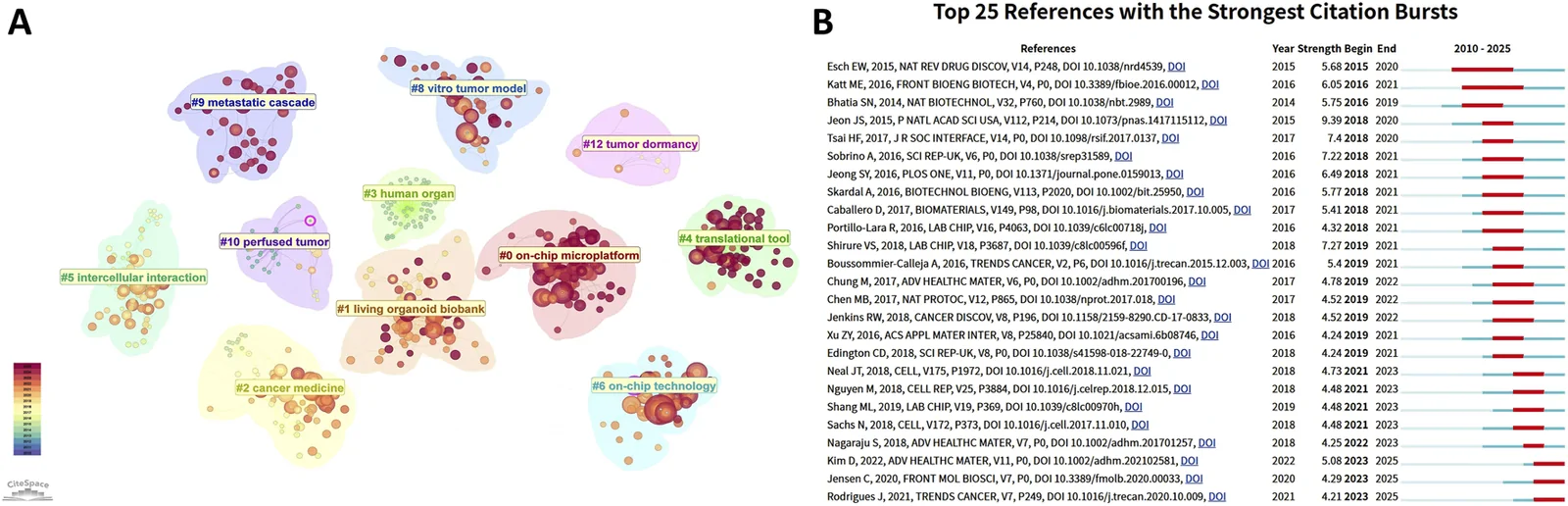
Reference co-citation clustering and citation-burst analysis in MPS and tumor-on-chip research. **(A)** Clusters of key terms in references. **(B)** Top 25 references with the strongest citation burst.

The citation-burst analysis in Figure 8B showed that attention to the field emerged in several successive waves. Jeon et al. (2015) had the strongest burst, followed by Tsai et al. (2017), Shirure et al. (2018), and Sobrino et al. (2016). Earlier bursts were mainly associated with organ-on-chip systems, vascularized tumor models, microfluidic platforms, and cancer-cell interactions. More recent bursts included references published by Kim et al. (2022), Jensen et al. (2020), and Rodrigues et al. (2021), whose burst periods extended into 2025. This temporal distribution suggests that the intellectual focus of the field has gradually broadened from the construction and validation of microfluidic platforms toward tumor microenvironment modeling, translational applications, and more biologically complex model systems. Citation bursts indicate periods of increased attention within the literature, but they should not be interpreted as direct measures of methodological superiority or clinical importance.

### 3.6 Keyword co-occurrence, thematic clustering, and temporal evolution

Keyword-based analyses were performed to characterize the thematic structure and temporal evolution of MPS and tumor-on-chip research using VOSviewer and CiteSpace. The co-occurrence and density maps (Figures 9A,B) showed that the field was organized around several closely related themes. “Tumor microenvironment,” “microfluidics,” “human,” “cancer,” and “microphysiological systems” occupied relatively central positions in the network, while terms such as “drug screening,” “organ-on-a-chip,” “tumor-on-a-chip,” “organoids,” “fibroblasts,” and “angiogenesis” linked engineering concepts with tumor biology and therapeutic applications. The high frequencies of “tumor microenvironment” (140 occurrences), “microfluidics” (114 occurrences), and “human” (94 occurrences) further indicate that the literature has focused on reconstructing biologically relevant tumor models under controlled microfluidic conditions.

**FIGURE 9:**
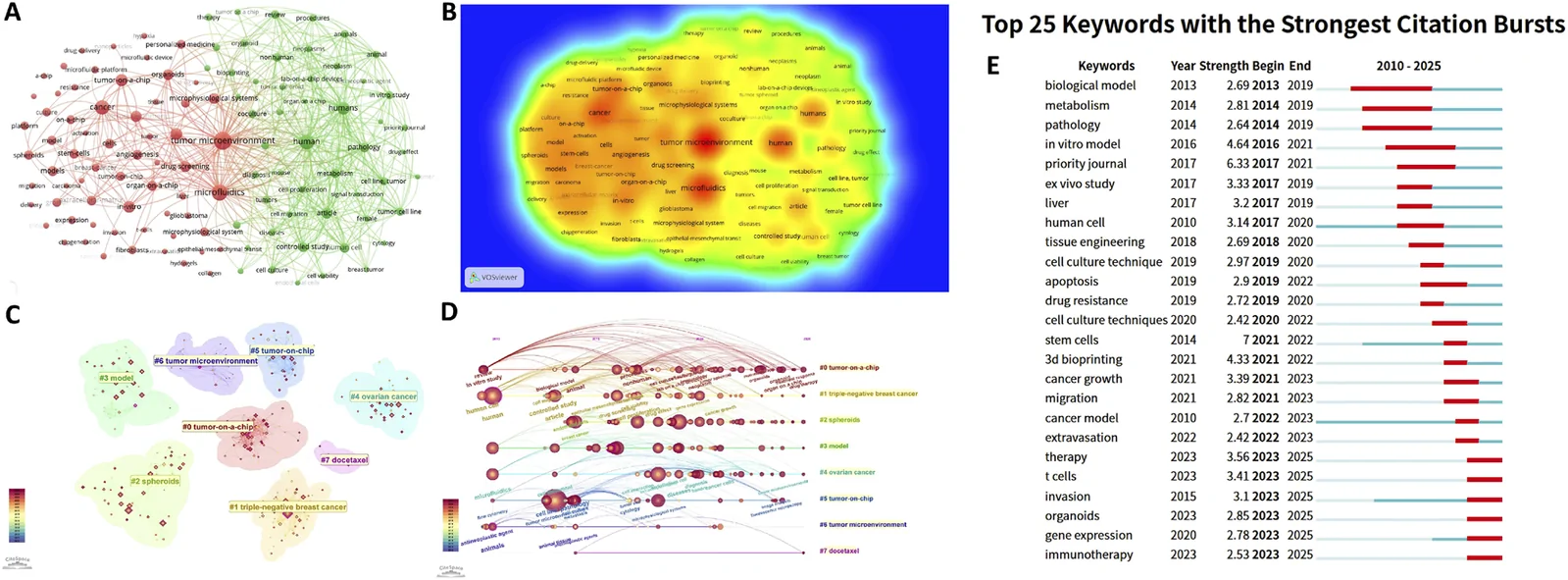
Keyword co-occurrence, thematic clustering, and temporal evolution in MPS and tumor-on-chip research. **(A)** Co-occurrence network map of keywords. **(B)** Density map of keywords. **(C)** Keywords clusters of all literature. **(D)** Timeline view of keywords clusters. **(E)** Top 25 keywords with strongest citation bursts.

Keyword clustering and timeline analysis (Figures 9C,D) provided a more detailed view of the thematic organization of the field. The main clusters were related to tumor-on-a-chip platforms, spheroids, tumor microenvironment, *in vitro* tumor models, ovarian cancer, triple-negative breast cancer, and docetaxel. These clusters show that the literature includes both general platform development and disease- or treatment-specific applications. The timeline view suggests that engineering terms and basic *in vitro* model concepts appeared throughout the earlier literature, whereas tumor microenvironment-related terms, organoid-related studies, and treatment-oriented topics became more visible in the later period.

The burst analysis in Figure 9E showed several changes in the topics receiving increased attention over time. Earlier bursts were associated with “biological model,” “metabolism,” “pathology,” “*in vitro* model,” “*ex vivo* study,” “liver,” and “human cell.” Subsequent bursts involved “tissue engineering,” “cell culture technique,” “apoptosis,” “drug resistance,” “stem cells,” and “3D bioprinting.” In the most recent period, keywords such as “cancer growth,” “migration,” “cancer model,” “extravasation,” “therapy,” “T cells,” “invasion,” “organoids,” “gene expression,” and “immunotherapy” showed increased citation activity. This progression is consistent with a gradual broadening of research interests from model construction and basic biological characterization toward tumor behavior, treatment response, immune-related interactions, and organoid-integrated applications.

Taken together, the keyword results suggest that cancer MPS and tumor-on-chip research has developed around the interaction of microfluidic engineering, tumor microenvironment reconstruction, and therapeutic evaluation. The increasing appearance of organoids, T cells, immunotherapy, drug resistance, and three-dimensional bioprinting in the later burst profile points to a growing interest in more complex and clinically relevant model systems. These keyword patterns describe changes in research attention and thematic emphasis; they do not, by themselves, establish that the newer platforms have achieved superior predictive performance or clinical utility. To examine the temporal differences in greater detail, a phase-split keyword analysis was subsequently conducted for the periods 2010–2020 and 2021–2025.

### 3.7 Phase-split keyword analysis

To further examine the temporal differences suggested by the overall keyword analysis, the study period was divided into an earlier phase from 2010 to 2020 and a later phase from 2021 to 2025. This division was used as an analytical framework for comparing changes in research emphasis rather than as a strict boundary between two completely separate stages of development. The comparison focused on the appearance of different model types, engineering approaches, biological processes, and translational applications.

During the research exploration phase (2010–2020), the clusters shown in Figure 10A were mainly related to nanotechnology, three-dimensional microfluidics, microfluidic devices, three-dimensional models, cancer in microfluidics, drug resistance, and tumor-on-chip systems. The timeline view in Figure 10B shows that these topics developed alongside terms related to human cells, controlled studies, cell lines, microfluidic analysis, and tumor models. The burst analysis in Figure 10C further identified “cell line,” “tumor cell line,” “breast neoplasms,” “endothelial cells,” “stem cells,” “coculture,” and “cell interaction” as topics that received increased attention during this period. Terms such as “personalized medicine,” “*ex vivo* study,” “drug effect,” and “cancer therapy” also appeared in the later part of the early phase, suggesting that researchers were beginning to connect platform development with treatment-related questions.

**FIGURE 10:**
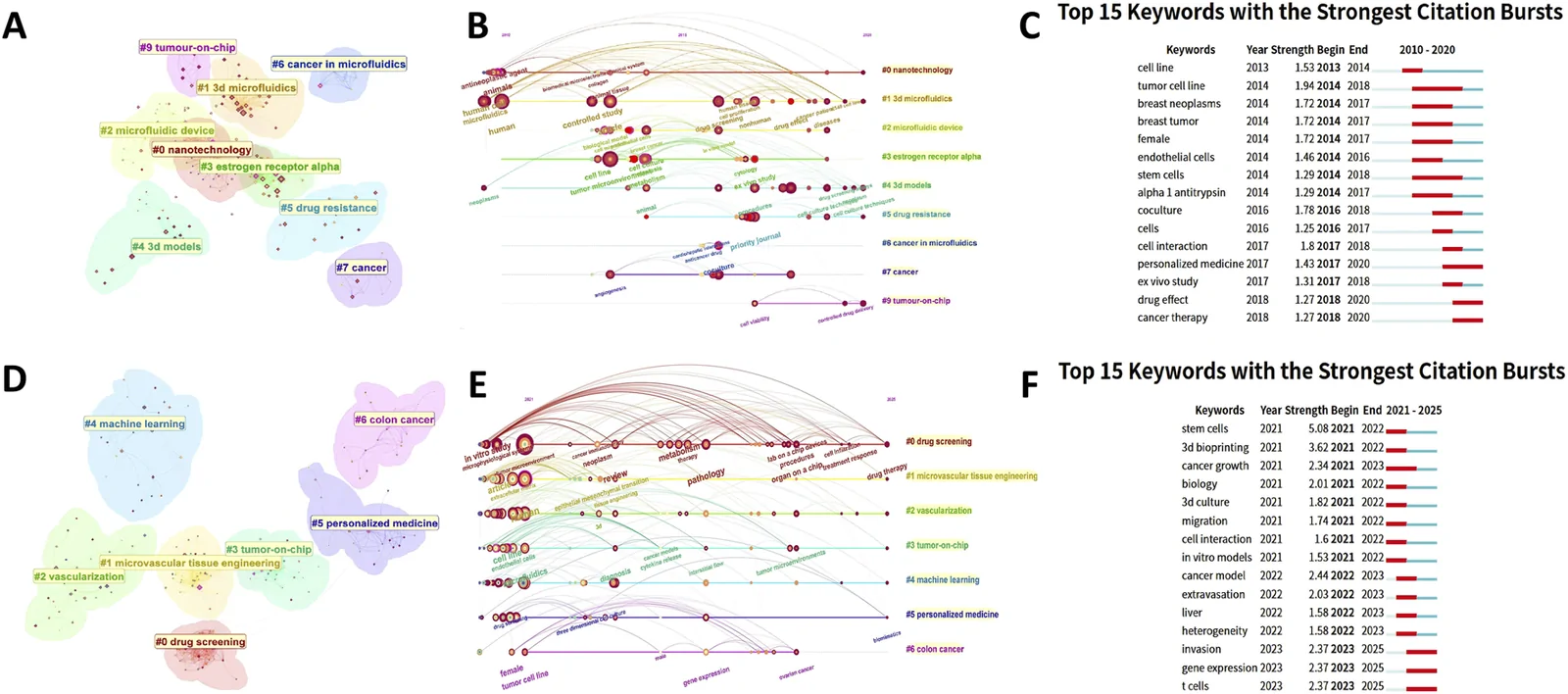
Phase-split keyword analysis of MPS and tumor-on-chip research. **(A)** Keywords clusters in research exploration phase. **(B)** Timeline view of keywords clusters in research exploration phase. **(C)** Top 15 keywords with strongest citation bursts in research exploration phase. **(D)** Keywords clusters in later expansion phase. **(E)** Timeline view of keywords clusters in later expansion phase. **(F)** Top 15 keywords with strongest citation bursts in later expansion phase.

The later expansion phase (2021–2025) showed a broader and more application-oriented cluster structure (Figure 10D). The main clusters included drug screening, microvascular tissue engineering, vascularization, tumor-on-chip systems, machine learning, personalized medicine, and colon cancer. In the timeline view (Figure 10E), these topics were connected with terms such as *in vitro* study, tumor microenvironment, metabolism, pathology, organ-on-a-chip, drug therapy, and extracellular matrix. This pattern suggests that recent research has increasingly addressed the biological context in which tumor models are studied, while also incorporating quantitative analysis, vascular components, and disease-specific applications.

The citation burst analysis for 2021–2025 (Figure 10F) showed strong recent activity around “stem cells,” “3D bioprinting,” “cancer growth,” “3D culture,” “migration,” “cell interaction,” and “*in vitro* models.” Other recent bursts included “cancer model,” “extravasation,” “heterogeneity,” “invasion,” “gene expression,” and “T cells,” with several of these topics remaining active through 2025. Compared with the earlier phase, the later burst profile contains more terms related to tumor behavior, multicellular interactions, vascular and immune processes, and functional characterization. The appearance of personalized medicine, machine learning, and drug screening in the later clusters also indicates growing interest in applying these platforms to treatment evaluation and patient-relevant modeling.

## 4 Discussion

### 4.1 Current status of the research field and international collaboration

The publication pattern observed in this study indicates that MPS and tumor-on-chip research has moved beyond its early exploratory stage and has become an increasingly active and interdisciplinary area. Publication output remained low before 2018 but increased considerably during the later years, reaching 101 articles in 2025. Publications from 2021 to 2025 accounted for 77.1% of the final corpus, suggesting that the recent expansion of the field has been driven by the convergence of microfluidic engineering, biomaterials, tumor biology, and translational modeling. The pattern does not point to a single defining breakthrough. Instead, it is more consistent with the gradual development of several related research directions that have increasingly been studied within the same platform-oriented framework.

The country-level analysis showed a clear concentration of research activity in the United States and China. The United States produced 148 articles, followed by China with 70 articles and South Korea with 38 articles. Italy, the United Kingdom, France, and the Netherlands also made visible contributions, although their publication outputs were lower. The collaboration structure shown in Figure 6 indicates that the United States and China occupied central positions in the international network, while European countries were connected with a broader range of partners. These results suggest that the two leading countries have played important but not identical roles in the development of the field: the United States has maintained the largest publication base and broad international connectivity, whereas China has established a rapidly expanding research presence in recent years.

The SCP and MCP distribution provides additional information about the regional organization of the literature. Publications from the United States and China were mainly single-country studies, whereas countries such as France, the Netherlands, Spain, Australia, and Iran showed a relatively higher proportion of multiple-country publications. This difference may reflect variation in research organization and international partnership patterns rather than a simple difference in scientific contribution. At the affiliation-entity level, the University of California System had the highest publication output, followed by Institut Curie and the University of Pittsburgh. Other highly productive entities included the Pennsylvania Commonwealth System of Higher Education, Harvard University, UNICANCER, and the Centre National de la Recherche Scientifique (Table 2). Because these entities were retained according to the original database affiliation strings, the institutional results describe indexed affiliation patterns and should not be interpreted as a fully standardized ranking of independent universities.

Countries such as the United Kingdom, France, the Netherlands, Italy, and Portugal occupied intermediate positions in terms of publication volume but remained well connected to the wider collaboration network. Their contribution appears to be reflected not only in the number of articles produced but also in their participation in cross-country research. Overall, the regional analysis points to an uneven but interconnected research landscape, with publication activity concentrated in North America and East Asia and collaboration links extending across Europe and other regions. The next part of the discussion therefore examines how the thematic focus of the field has changed across the earlier and later phases of its development.

### 4.2 Interpretation of phase-specific thematic evolution

The phase-split analysis suggests a gradual change in thematic emphasis rather than an abrupt transition from engineering to biology. The earlier literature established the microfluidic and three-dimensional modeling framework, while the later literature increasingly incorporated vascularization, complex cell interactions, tumor heterogeneity, drug screening, and patient-oriented applications. Organoids and related three-dimensional models appeared as part of this later expansion. The observed changes should therefore be interpreted as shifts in research attention within the broader cancer MPS and tumor-on-chip field.

## 5 Limitations

The present study should be interpreted in light of several limitations related to corpus construction and bibliometric inference. The initial search strategy was intentionally broad to maximize retrieval sensitivity and included terms referring to tumor and cancer models, tissue-on-chip, tumor-on-chip, microphysiological systems, and three-dimensional microfluidic models. Because these terms do not necessarily imply organoid integration, a predefined secondary title and abstract screening framework was applied after the initial screening. Of the 800 records retained at that stage, 324 were excluded because they did not meet the final scope, leaving 476 publications for analysis. This additional screening improved the alignment between the dataset and the stated research scope, but it cannot eliminate all classification uncertainty. Eligibility was determined from the information available in titles and abstracts, and some relevant studies may have used terminology that was not captured by the search strategy. The final corpus should therefore be understood as a structured representation of MPS and tumor-on-chip research, in which organoid-integrated models constitute an emerging component rather than a requirement for every included publication.

Only English-language publications were included, which may have introduced language and regional bias. The use of bibliometric software may also have affected the recognition of author names, institutional affiliations, and other bibliographic metadata across databases.

Citation-based indicators are also influenced by the time available for a publication to accumulate citations. Earlier studies generally have a longer citation window, whereas recent publications may appear less influential simply because they have been available for a shorter period. Therefore, the higher citation averages observed for earlier years and the lower values observed for recent years should not be interpreted as direct measures of differences in research quality. Journal-level indicators, such as impact factor, should likewise not be regarded as substitutes for evaluating the quality or translational value of individual articles.

Manual curation was limited to checking duplicate records and verifying DOI and title information during data preprocessing. Citation counts, collaboration networks, keyword clusters, and citation burst results were generated from the processed dataset without further subjective adjustment. However, differences in author names, institutional affiliations, and other bibliographic metadata between WoSCC and Scopus may still affect how records are grouped and represented in the analyses.

Another limitation is that bibliometric analysis cannot directly evaluate the quality or biological performance of MPS and tumor-on-chip platforms. Bibliographic records rarely provide sufficiently detailed and consistent information on chip materials, cell sources, patient-derived models, immune or vascular components, experimental validation, and reproducibility. Therefore, the present analysis can identify research attention toward drug screening, organoids, or immunotherapy, but cannot determine whether a specific platform accurately predicts drug response or has demonstrated clinical utility.

## 6 Conclusion

Overall, this dual-database analysis of 476 publications published between 2010 and 2025 characterizes the development of MPS and tumor-on-chip research. Publication activity began to accelerate around 2018, and the phase-split analysis compared the earlier period from 2010 to 2020 with the later expansion period from 2021 to 2025. Compared with the earlier period, recent studies have shown greater attention to tumor microenvironment reconstruction, multicellular interactions, drug-related applications, and organoid-integrated models. Organoid integration should therefore be understood as an emerging component within the broader cancer MPS and tumor-on-chip field, rather than as a defining feature of all included studies. These findings describe changes in publication activity, knowledge structure, and research attention, but bibliometric patterns alone cannot establish the biological fidelity, drug-response predictive performance, reproducibility, or clinical utility of individual platforms. Further studies combining bibliometric monitoring with structured full-text assessment and experimental validation will be needed to determine which cancer MPS and tumor-on-chip configurations are most suitable for translational oncology.

## Data Availability

The original contributions presented in the study are included in the article/supplementary material, further inquiries can be directed to the corresponding authors.
